# Insight into the Thermal Washing Mechanism of Sodium Lignosulfonate Alkyl/Sodium Persulfate Compound on Oily Sludge

**DOI:** 10.3390/ijms252312542

**Published:** 2024-11-22

**Authors:** Yun Ma, Hui Liu, Liuli Zhu, Yi Xie, Chuanqi Ren, Xiaorong Mo, Xiaoying Liu, Chen Liang, Gang Deng, Shuangquan Yao, Chengrong Qin

**Affiliations:** 1 Guangxi Key Laboratory of Clean Pulp & Papermaking and Pollution Control, School of Light Industrial and Food Engineering, Guangxi University, Nanning 530004, China; 1916401003@gxu.edu.cn (Y.M.); 2316301027@st.gxu.edu.cn (H.L.); 2316391078@st.gxu.edu.cn (L.Z.); 2316301050@st.gxu.edu.cn (Y.X.); 2316391039@st.gxu.edu.cn (C.R.); 2216301036@st.gxu.edu.cn (X.M.); 2216301032@st.gxu.edu.cn (X.L.); liangchen@gxu.edu.cn (C.L.); 2 College of Biotechnology and Sericultural Research Institute, Jiangsu University of Science and Technology, Zhenjiang 212100, China; jayderek@foxmail.com

**Keywords:** sodium lignosulfonate alkyl, sodium persulfate, oily sludge, oxidation mechanism

## Abstract

The thermal washing of oily sludge using sodium persulfate (SD) assisted by sodium lignosulfonate surfactant has been demonstrated to be an effective method for oily sludge remediation. To further explore the underlying mechanisms of this process, a systematic study was conducted by simulating oily sludge systems consisting of saturated hydrocarbons (SaH), aromatics hydrocarbons (ArH), resins (Res), and asphaltenes (Asp). The effects of reaction conditions, such as pH, sodium lignosulfonate alkyl (LSA) concentration, SD concentration, and washing temperature, were analyzed. Furthermore, the oxidative kinetic mechanism during the reaction process was investigated. The results demonstrated that neither petroleum hydrocarbons nor SD underwent significant chemical transformations when exposed to LSA, while SD exhibited a marked oxidative degradation effect on all four types of hydrocarbons. Oxidation kinetics indicated that sodium hydroxide played a catalytic role, with SD being the main oxidant and particularly efficient in degrading Asp and Res. Meanwhile, LSA contributed to the removal of hydrocarbons by reducing the surface tension of the solution, enhancing solubilization. This study not only elucidates the central role of SD in the thermal washing process but also provides a solid theoretical foundation for the practical application of this technology in oily sludge treatment.

## 1. Introduction

Oily sludge, a complex waste byproduct generated during petroleum extraction, transportation, and refining, consists primarily of heavy crude oil fractions, water, and sediments. Its high viscosity and low permeability present significant challenges for effective treatment [1]. In addition to its substantial oil content, oily sludge has also harbored toxic and hazardous compounds, including benzene derivatives and phenols, which have already posed significant environmental hazards [2]. Consequently, the efficient treatment and resource recovery of oily sludge have emerged as critical research priorities in the field of environmental protection.

Currently, the treatment of oily sludge primarily involves chemical, physical, and biological methods; among these, chemical methods have gained prominence in practical applications due to their high efficiency [3]. Surfactants, widely utilized as amphiphilic agents in thermal washing, are employed to enhance the diffusion and mobility of petroleum hydrocarbons and other organic contaminants within soil matrices, thereby facilitating the efficient separation of oil and water phases in oily sludge [4]. Nevertheless, surfactant-based thermal washing technologies present several limitations. First, the process can generate secondary pollutants in both the soil and washing solutions, potentially leading to further environmental contamination if not adequately controlled. Second, the efficiency of surfactant-based washing remains insufficient for the treatment of heavy petroleum hydrocarbons [5]. In recent years, thermal washing technologies that integrate surfactants with oxidation processes have emerged as a key area of research in oily sludge remediation [6]. These methods primarily utilize oxidants or other electron transfer techniques to break down high-molecular-weight organic compounds into low-molecular-weight substances [7], thereby enhancing the degradation and recovery of petroleum hydrocarbons. This integrated approach is recognized as a novel and promising strategy for oily sludge treatment. However, the combined use of surfactants and oxidants raises concerns regarding potential soil and water contamination [8,9]. Several studies have investigated this integrated approach. For instance, Trellu et al. [10] demonstrated that the use of nonionic surfactant Tween 80 combined with anodic oxidation could selectively degrade petroleum hydrocarbons, such as polycyclic ArH hydrocarbons, in soil, enhancing the solubilization of PAHs by the surfactant. However, micelle formation negatively impacted the anodic oxidation process. Similarly, Huguenot et al. [11] reported that combining Tween 80 with Fenton oxidation could completely mineralize hydrocarbons in diesel-contaminated soil, significantly enhancing diesel solubilization. However, they also observed an increase in the toxicity of the washing effluent over time. Given these challenges, it is imperative to develop a non-toxic, highly efficient oxidation degradation method that can address current environmental pollution issues associated with oily sludge treatment.

Sodium lignosulfonate, a natural polymeric anionic surfactant, exhibits excellent dispersibility, stability, and surface activity, along with the advantages of wide availability, biodegradability, and low cost [12]. It is considered a promising green surfactant. Sodium persulfate, widely used as an oxidant in in situ chemical oxidation (ISCO) technologies, generates highly reactive oxidative radicals, such as SO_4_^−^• and •OH, upon activation, which effectively degrade petroleum-based pollutants [13]. This oxidant has been extensively applied for the remediation of petroleum-contaminated soils globally [9]. In our previous studies, we prepared a series of combined surfactants by varying the mass ratio of sodium persulfate to sodium lignosulfonate and used them as thermal washing agents for the treatment of oily sludge. The experimental results demonstrated that, compared to sodium lignosulfonate alone, the combined surfactants significantly enhanced oil removal efficiency during thermal washing [6]. However, the synergistic mechanism of oil removal between sodium persulfate and sodium lignosulfonate remains unclear and warrants further investigation.

Based on the aforementioned analysis, sodium persulfate and sodium lignosulfonate alkyl were selected as reagents for the thermal washing of oily sludge. Kinetic desorption studies were performed on four representative simulated oily sludge components: SaH, ArH, Res, and Asp. A systematic investigation was conducted to evaluate the effects of pH, sodium lignosulfonate concentration, sodium persulfate concentration, and system temperature on the desorption process, with sodium persulfate serving as a control variable. The oxidative kinetic mechanism was further explored during the reaction. Infrared spectroscopy was employed to analyze the interactions among the components, and the reaction mechanism was validated through multidimensional analyses, including surface tension measurements, HLB values, foam ability, and pH changes. The results provide essential theoretical support and practical guidance for optimizing the oily sludge treatment process. See the end of the document for further details on references.

## 2. Results and Discussion

### 2.1. Oxidation Effects of Oil Components During Thermal Washing Processes

#### 2.1.1. Effect of pH on the Removal Rate of Petroleum Hydrocarbons

**Figure 1:**
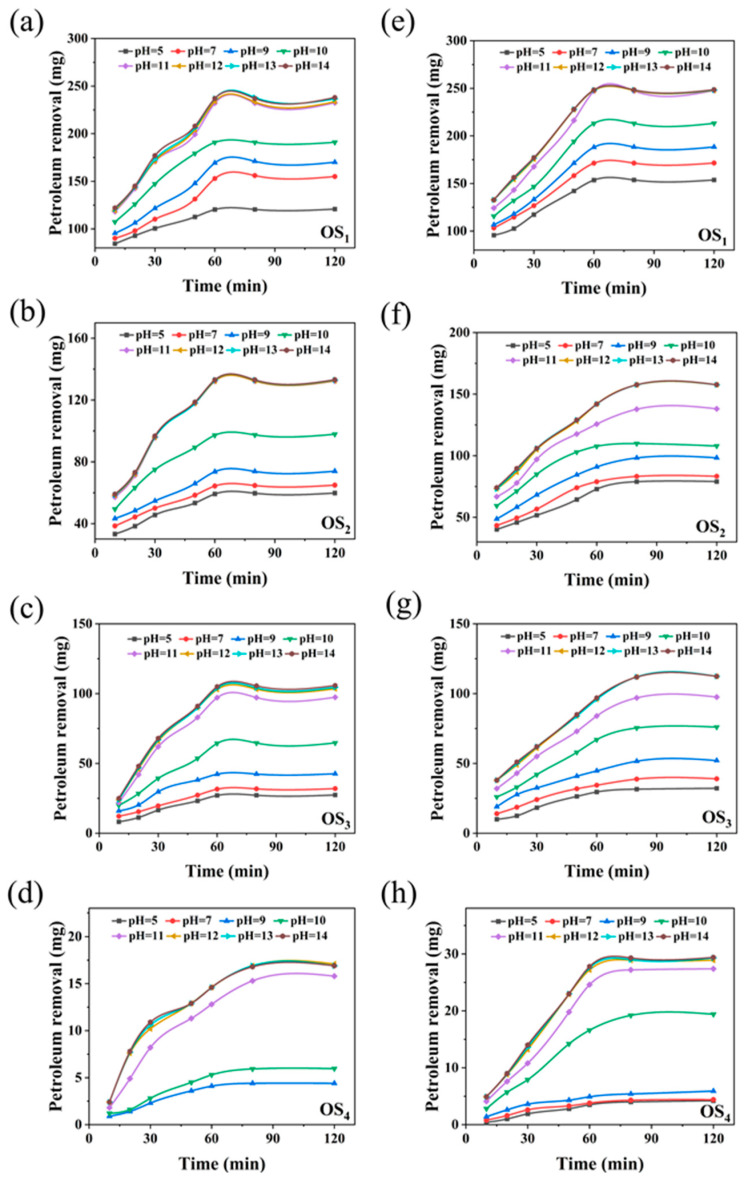
The effect of pH value on the removal of oil from four simulated oily sludge by hot washing with LSA/SD ((**a**–**d**) without SD; (**e**–**h**) with SD. OS_1_: SaH sludge; OS_2_: ArH sludge; OS_3_: Res sludge; OS_4_: Asp sludge).

To investigate the effect of pH on the removal efficiency of petroleum hydrocarbons under the synergistic action of SD and LSA, a reaction kinetics model was established to analyze the degradation rate of petroleum hydrocarbons at different pH levels. The experiments were conducted under optimal thermal washing conditions [6]: 2.0 g/L sodium lignosulfonate alkylate, 5.0% sodium persulfate, temperature of 40 °C, liquid-to-solid ratio of 25:1, and stirring speed of 150 rpm. The pH was adjusted between 5 and 14, and thermal washing was performed on four types of simulated oily sludge. Samples were taken at regular intervals to determine the petroleum hydrocarbon content. A control group without sodium persulfate was included. Figure 1 shows the variation trends in petroleum hydrocarbon removal under different pH values and at different times, both in the presence and absence of sodium persulfate.

As shown in Figure 1a–h, a clear upward trend in the removal of petroleum hydrocarbons from the four simulated oily sludges (SaH, ArH, Res, and Asp) is observed with increasing pH. Notably, when the pH reaches 11, the removal rate of SaH tends to plateau. At this point, the addition of 5.0% SD (Figure 1e) does not change the pH at the saturation point, and the increase in removal efficiency is only 5.8%. This indicates that the removal of SaH is relatively insensitive to pH changes. In contrast, as shown in Figure 1f–h, the pH required to achieve the maximum removal of ArH, Res, and Asp increases significantly from 11 to 13. This suggests that the removal of heavier fractions, such as Res and Asp, requires a more stringent alkaline environment after the addition of SD. However, at pH 14, the removal rate of petroleum hydrocarbons slightly decreases. This is likely due to sulfate ions reacting with trace cations present in the soil, such as magnesium and calcium, forming precipitates that reduce the oxidative degradation efficiency of sulfate ions [14,15]. The removal rates of ArH, Res, and Asp increased by 18.5%, 12.2%, and 72.3%, respectively. These results strongly demonstrate a significant synergistic effect between SD and LSA, particularly in enhancing the removal of heavy fractions such as ArH, Res, and Asp, with the most pronounced effect observed for Asp. In contrast, the synergistic removal effect on SaH was relatively limited. Furthermore, from a reaction kinetics perspective, it was found that during thermal washing with LSA alone, the removal of SaH, ArH, and Res rapidly reached equilibrium within 60 min, while the removal of Asp was slower, requiring 80 min. However, upon introducing SD, although the equilibrium time for SaH remained unchanged, the equilibrium times for the removal of ArH, Res, and Asp were extended to 80 min. This change is likely due to the initial oxidation of petroleum hydrocarbons by SD, generating smaller hydrocarbon intermediates that are more easily emulsified by LSA [16], thereby prolonging the overall removal process to reach equilibrium.

Based on previous studies on the kinetics of oily sludge removal, both sides of Equation (19) were logarithmically transformed, resulting in Equation (1):

(1) $$\log\left(dw/dt\right)=\log\left({Ae}^{-\left(\frac{E}{RT}\right)}\times {\left[LSA\right]}^{b}\times {\left[SD\right]}^{C}\right)+a_{n}log\left({OH}^{-}\right)$$

**Figure 2:**
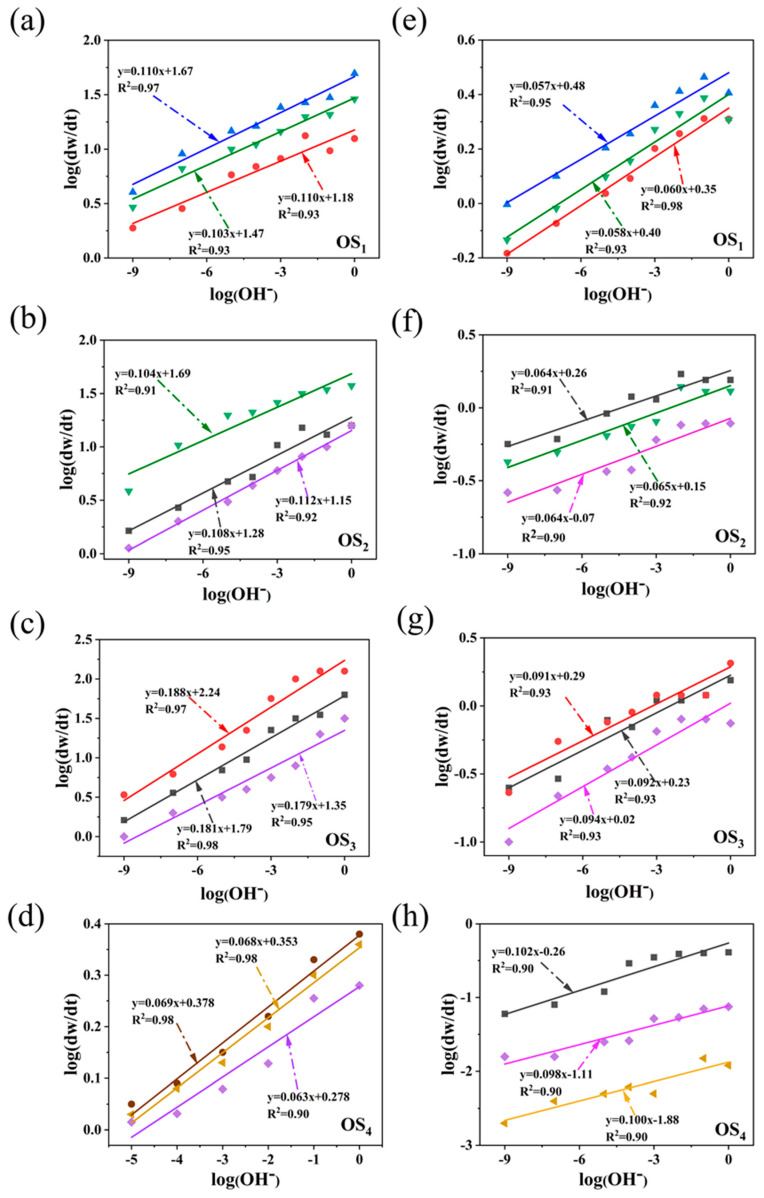
Linear relationship between log(d*w*/d*t*) and log(*OH^−^*) by hot washing with LSA/SD ((**a**–**d**) without SD; (**e**–**h**) with SD. OS_1_: SaH sludge; OS_2_: ArH sludge; OS_3_: Res sludge; OS_4_: Asp sludge).

As shown in Figure 2a–h, linear regression analysis was performed to determine the linear relationship between log (d*w*/d*t*) and log (*OH^−^*) for the four types of simulated oily sludge, both in the presence and absence of sodium persulfate.

In the linear relationship for the thermal washing process using LSA alone Figure 2a–d, the *a*_n_ values for the simulated oily sludge containing SaH, ArH, Res, and Asp were 0.110, 0.104, 0.188, and 0.068, respectively. The reaction order was positive in all cases, indicating a direct correlation with the reaction rate. The concentration of OH^−^ had a significantly greater effect on the removal of SaH, ArH, and Res compared to Asp during the oxidation process. This suggests that an alkaline environment is a key factor in enhancing the removal efficiency of SaH, ArH, and Res in the thermal washing system, whereas Asp removal is relatively less dependent on alkalinity. However, a notable change occurred in the thermal washing process when SD was introduced as a co-treatment with LSA. The *a*_n_ values for the SaH, ArH, and Res decreased to 0.058, 0.064, and 0.091, respectively, while the *a*_n_ value for the Asp-containing oily sludge increased to 0.102. The reduction in the *a*_n_ values for SaH, ArH, and Res indicates that after the addition of SD, the removal efficiency of these hydrocarbons was significantly improved even at lower pH levels [16], thereby reducing their dependence on an alkaline environment. This change suggests that the presence of SD promotes the oxidation reactions, making it easier to separate these hydrocarbons from the sludge.

Of particular note is the significant increase in the *a*_n_ value for Asp in this process. This may be due to the fact that the alkaline environment not only maintained but also enhanced the activation of sodium persulfate, generating more SO₄^−^• and •OH radicals. These strong oxidative radicals can effectively attack the large molecular structure of Asp, breaking them down into smaller hydrocarbon molecules, thereby increasing the removal efficiency of Asp [15,17]. As a result, the synergistic action of sodium persulfate not only optimized the removal conditions for SaH, ArH, and Res but also significantly enhanced the degradation and removal of Asp.

#### 2.1.2. Effect of LSA Concentration on the Removal Rate of Petroleum Hydrocarbons

**Figure 3:**
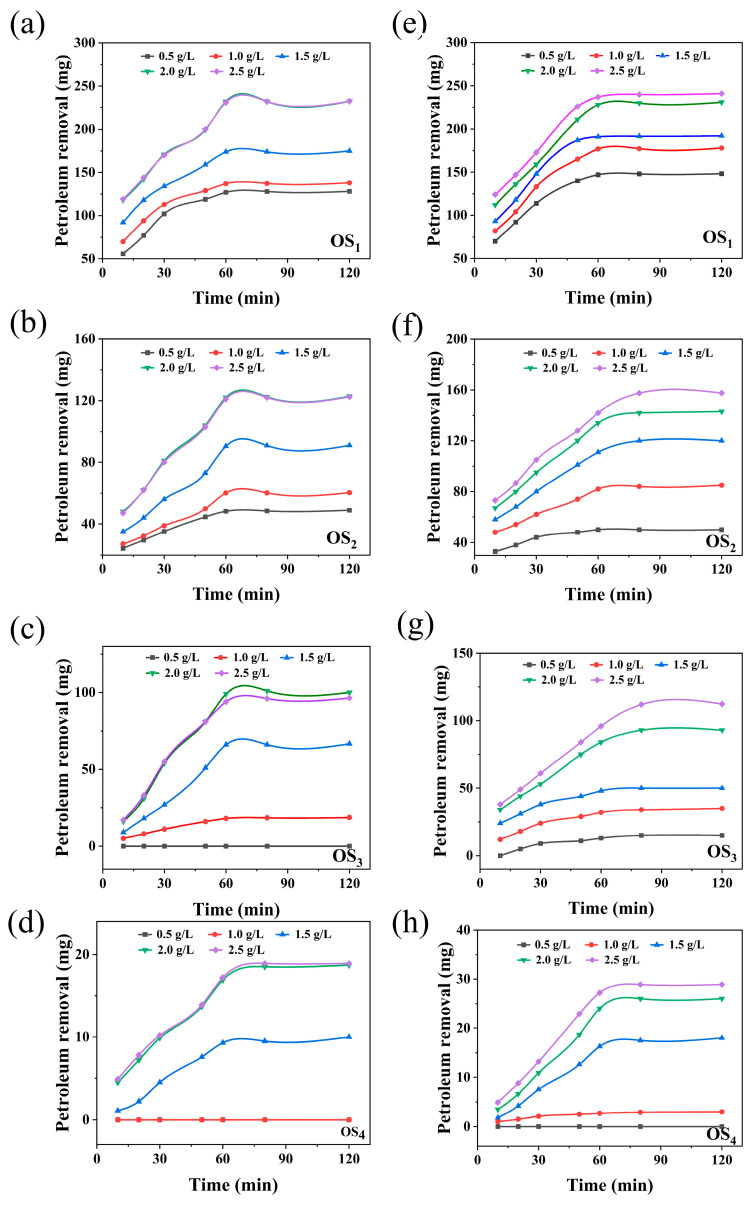
The effect of LSA concentration on the removal of oil from four simulated oily sludge by hot washing with LSA/SD ((**a**–**d**) without SD; (**e**–**h**) with SD. OS_1_: SaH sludge; OS_2_: ArH sludge; OS_3_: Res sludge; OS_4_: Asp sludge).

To investigate the effect of LSA concentration on petroleum hydrocarbon removal in the presence of SD, the pH systems of the four types of simulated oil sludge thermal washing fluids were all operated under optimal conditions; for SaH, the washing solution pH was maintained at 11, while for ArH, Res, and Asp, the pH was set at 13. All other parameters were kept under optimal experimental conditions [6]. The concentration of sodium lignosulfonate alkylate (LSA) was varied between 0.5 and 2.5 g/L. Samples were taken at regular intervals to determine the petroleum hydrocarbon content. A control group without the addition of SD was also included. Figure 3 illustrates the trends in oil removal at different LSA concentrations, both in the presence and absence of SD.

As shown in Figure 3a–f, the removal of petroleum hydrocarbons from all four types of simulated oily sludge increased with the rising concentration of LSA, both in the LSA thermal washing system (Figure 3a–d) and the LSA/SD thermal washing system (Figure 3e–h). In the LSA-only system (Figure 3a–d), the removal reached its maximum when the LSA concentration was 2.0 g/L. At this concentration, the maximum removal amounts for SaH, ArH, Res, and Asp were 232.152 mg, 122.314 mg, 98.96 1 mg, and 16.943 mg, respectively. This increase is attributed to the LSA reaching its critical micelle concentration (CMC) at 2.0 g/L [18], forming a stable oil-in-water emulsion. At this point, the surface tension of the solution reached its lowest value, and the interfacial tension between the petroleum hydrocarbons and sludge reached its maximum, thus further increasing the LSA concentration did not enhance the removal efficiency [19]. The removal efficiency followed the order: SaH > ArH > Res > Asp, indicating that LSA exhibits high efficiency in removing lighter oil components. This is due to the fact that, compared to aromatic hydrocarbons, resins, and asphaltenes, saturated hydrocarbons possess stronger non-polarity, rendering them the most hydrophobic. LSA, as an anionic surfactant, exhibits a higher affinity towards oil components with stronger non-polarity, enabling these components to more easily bind to the hydrophobic ends of LSA compounds. Consequently, saturated hydrocarbons are more readily encapsulated by LSA solutions to form micelles, resulting in the highest removal efficiency for saturated hydrocarbons. while its effectiveness for heavier oil fractions is limited. When 5.0% SD was introduced into the system (Figure 3e–h), the effective concentration threshold of LSA increased to 2.5 g/L. At this concentration, the removal of all four types of oily sludge significantly improved, with the removal rates of SaH, ArH, Res, and Asp increasing by 3.5%, 28.8%, 13.5%, and 70.4%, respectively. This demonstrates that the addition of SD significantly enhances the oxidative degradation of ArH, Res, and Asp, with the most notable effect observed for Asp [20]. In contrast, the improvement in the removal of SaH was relatively small due to their ease of emulsification [21]. Additionally, SD may interact with LSA molecules, indirectly influencing its emulsification efficiency, resulting in the optimal concentration of LSA shifting to 2.5 g/L [22,23]. In terms of reaction kinetics, the LSA-only system reached equilibrium for the removal of SaH, ArH, and Res within 60 min, whereas the emulsification of Asp required 80 min, reflecting the greater difficulty in emulsifying Asp. However, in the LSA/SD system, SaH, which are less affected by oxidation and easier to emulsify, still reached equilibrium within 60 min. Asp, benefiting from oxidative degradation and the generation of smaller, more easily emulsified molecules [9], also had their equilibrium time reduced to 60 min. In contrast, the equilibrium time for ArH and Res extended to 80 min, as the degradation of complex macromolecules produced more intermediates, requiring additional time to form a stable emulsion system.

Based on previous studies on the kinetics of oily sludge removal [24], the logarithm was applied to both sides of Equation (18), yielding Equation (2):

(2) $$\log\left(\frac{dw}{dt}\right)=\log\left({Ae}^{-\left(\frac{E}{RT}\right)}\times {\left[{OH}^{-}\right]}^{a}\times {\left[SD\right]}^{C}\right)+b_{n}log\left(LSA\right)$$

**Figure 4:**
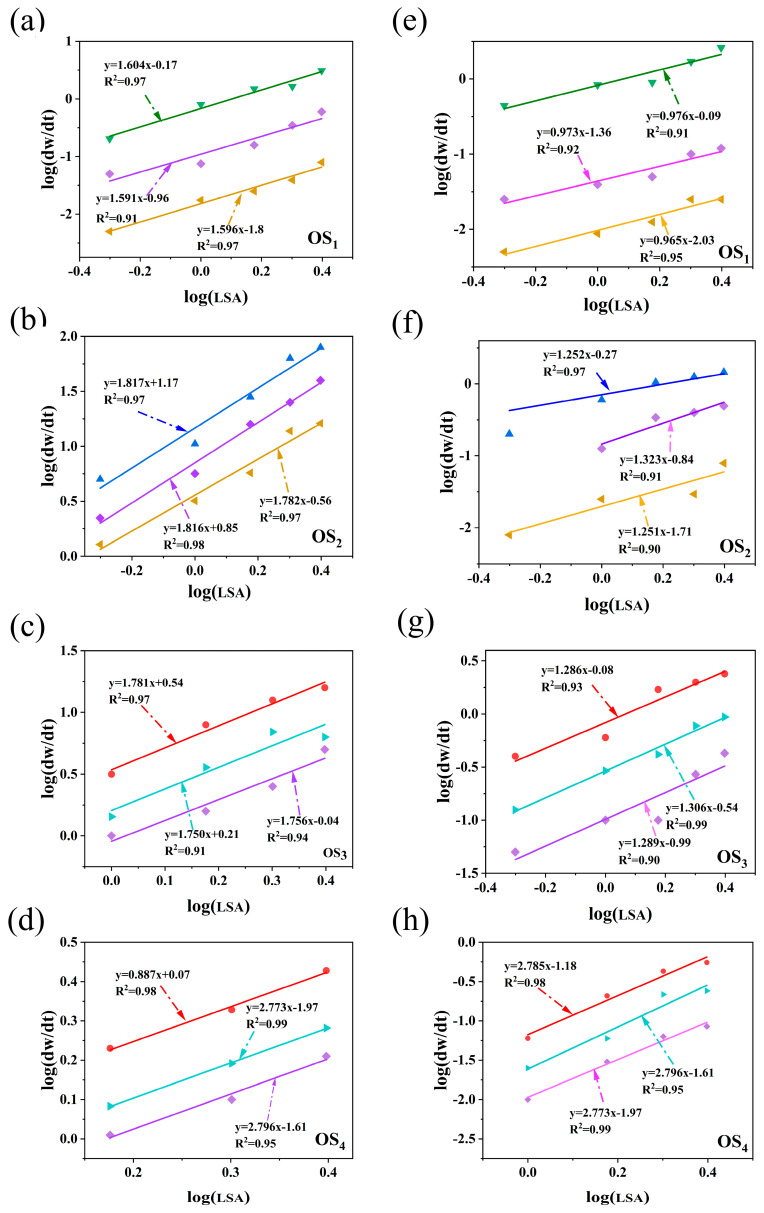
Linear relationship between log (d*w*/d*t*) and log [*LSA*] by hot washing with LSA/SD ((**a**–**d**) without SD; (**e**–**h**) with SD. OS_1_: SaH sludge; OS_2_: ArH sludge; OS_3_: Res sludge; OS_4_: Asp sludge).

As shown in Figure 4a–h, the linear relationship between log(d*w*/d*t*) and log(*LSA*) for the four types of simulated oily sludge, both in the presence and absence of SD, was obtained through linear regression analysis.

**Figure 5:**
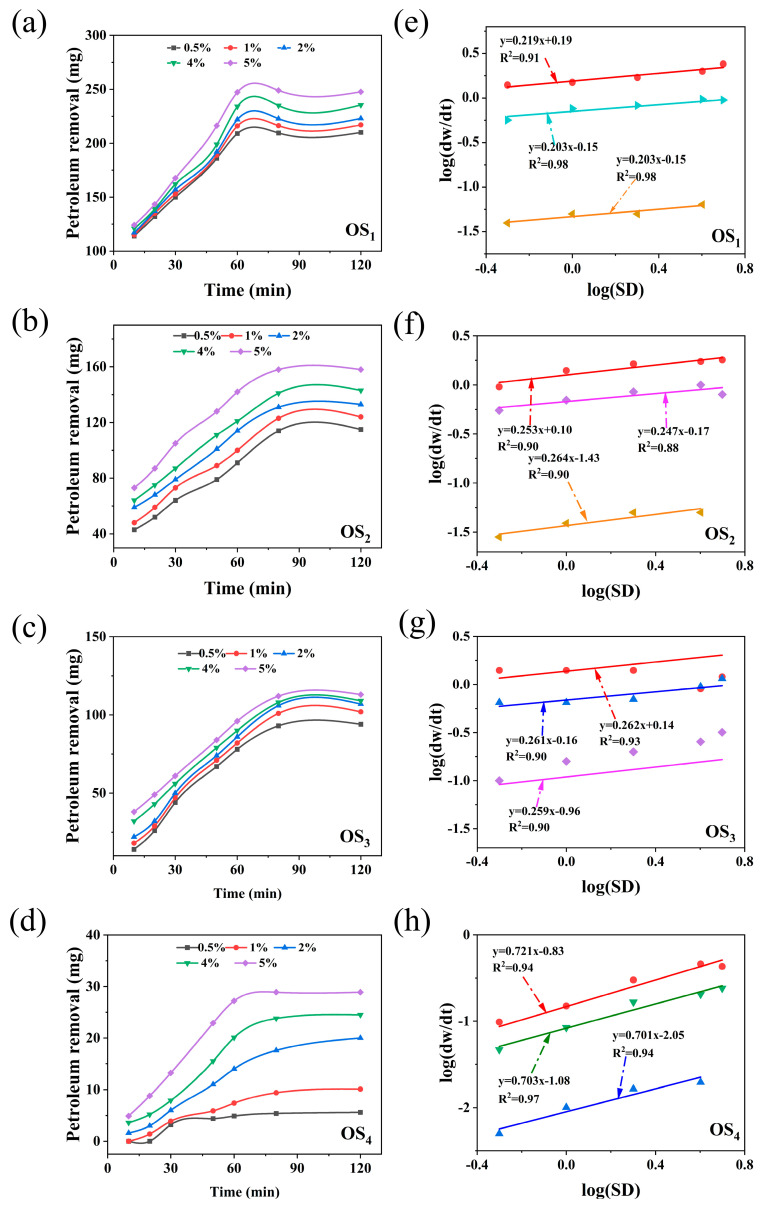
The effect of SD concentration on the removal of oil from four simulated oily sludge and linear relationship between log(d*w*/d*t*) and log[*LSA*] by hot washing with LSA/SD ((**a**–**d**) oil removal effect.; (**e**–**h**) linear relationship. OS_1_: SaH sludge; OS_2_: ArH sludge; OS_3_: Res sludge; OS_4_: Asp sludge).

The linear relationship for the LSA-only washing process is shown in Figure 4a–d. For the simulated oily sludge containing SaH, ArH, Res, and Asp, the *b*_n_ values are 1.591, 1.779, 1.784, and 2.772, respectively. This trend clearly indicates that as the complexity and difficulty of the treated components increase, the *b*_n_ values rise accordingly. During the removal of petroleum hydrocarbons using LSA solutions, the removal of Asp presents the greatest challenge, whereas the removal of SaH, ArH, and Res is relatively easier. The linear relationship for the LSA/SD washing process is illustrated in Figure 5e–h, where the *b*_n_ values for SaH, ArH, Res, and Asp are 0.971, 1.247, 1.282, and 2.779, respectively. Compared with the LSA-only washing process, the *b*_n_ values for SaH, ArH, and Res show a slight decrease, while the *b*_n_ value for Asp remains nearly unchanged. This phenomenon strongly demonstrates the chemical stability of LSA, suggesting that its primary role in the washing process is more inclined toward physical separation rather than significant chemical changes. This finding provides valuable insight for optimizing the washing process and enhancing the efficiency of oily sludge treatment, etc.

#### 2.1.3. Effect of SD Concentration on the Rate of Petroleum Hydrocarbon Removal

To investigate the influence of SD concentration on the removal efficiency of petroleum hydrocarbons during the synergistic thermal washing of oily sludge with LSA and SD, a kinetic model was developed to characterize the degradation of petroleum hydrocarbons at varying SD concentrations. The LSA concentration was maintained at 2.5 g/L, with all other parameters optimized according to the established experimental conditions. Figure 5a–f illustrates the trends in petroleum hydrocarbon removal and the corresponding linear relationships for four types of simulated oily sludge across different sodium persulfate concentrations.

As shown in Figure 5a–d, a clear increasing trend in petroleum hydrocarbon removal was observed in all four types of simulated oily sludge with the increase in SD concentration. The removal efficiency was most significant when the SD dosage reached 5.0%, with the removal amounts of SaH, ArH, Res, and Asp reaching 247.333 mg, 158.271 mg, 112.620 mg, and 28.938 mg, respectively. Further analysis revealed that the removal of saturates reached equilibrium within 60 min, indicating a relatively fast oxidation and degradation rate. This may have facilitated the rapid emulsification of the small molecular components of saturates in the LSA solution. In contrast, the removal of ArH, Res, and Asp was more time-consuming, reaching equilibrium within 80 min. At lower SD concentrations, the degradation of ArH, Res, and Asp was more challenging, especially for the asphaltene fraction, where the degradation process not only took longer but also exhibited lower efficiency, with a significantly slower reaction rate compared to higher SD concentrations [8]. These findings reveal the differential impact of SD concentration on the degradation characteristics of various petroleum hydrocarbon fractions.

Based on previous studies on the kinetics of oily sludge removal, logarithmic transformation was applied to both sides of Equation (1), yielding Equation (3):

(3) $$\log\left(\frac{dw}{dt}\right)=\log\left({Ae}^{-\left(\frac{E}{RT}\right)}\times {\left[{OH}^{-}\right]}^{a}\times {\left[LSA\right]}^{b}\right)+c_{n}log\left(SD\right)$$

Linear regression analysis was used to determine the linear relationships between log (d*w*/d*t*) and log (*SD*) for the four types of simulated oily sludge during the LSA/SD synergistic reaction, as shown in Figure 5e–h. The values of *c*_n_ (reaction order) increased with the difficulty of treatment.

The *c*_n_ values for saturates, ArH, Res, and Asp were 0.201, 0.254, 0.261, and 0.704, respectively, indicating that SD is more effective in promoting oxidation and degradation of heavier components. As the molecular weight and compositional complexity of petroleum hydrocarbons increase, the oxidative degradation intensity of SD in the thermal washing process is enhanced.

#### 2.1.4. Effect of Reaction System Temperature on the Petroleum Hydrocarbon Removal Rate

**Figure 6:**
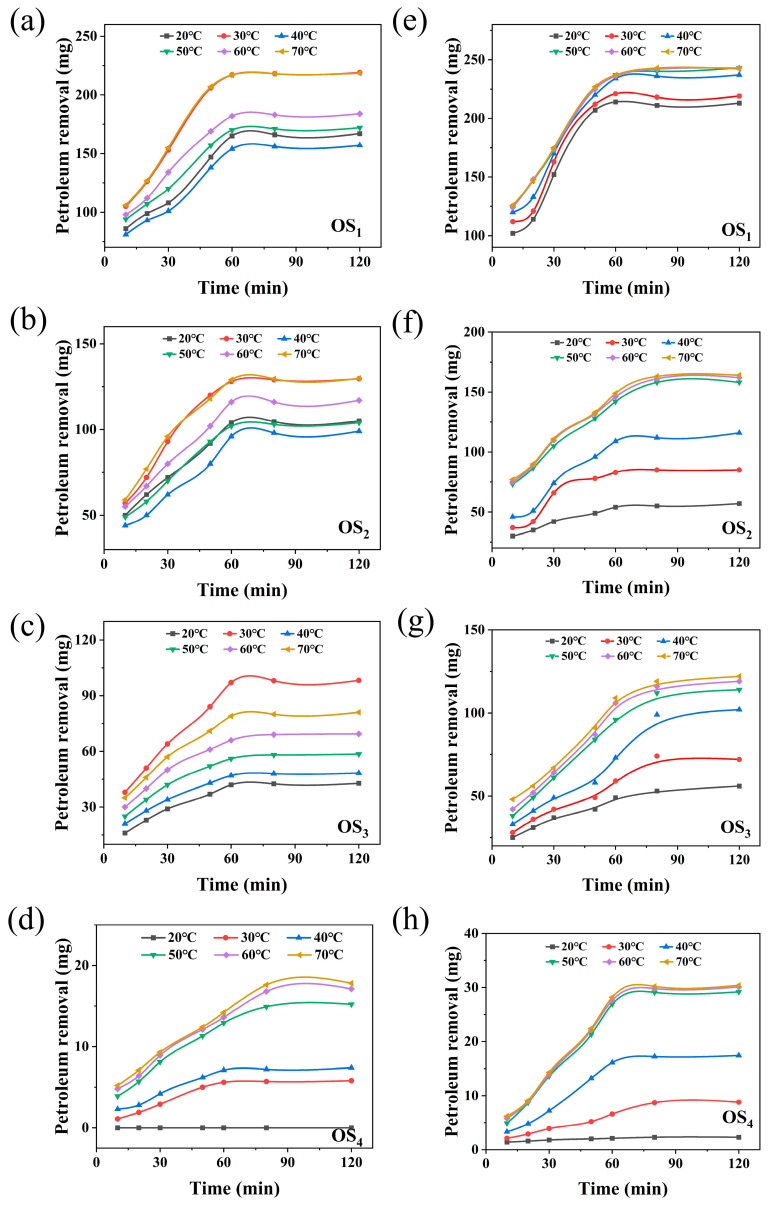
Effect of temperature on removal of oil from four simulated oily sludge by hot washing with LSA/SD ((**a**–**d**) without SD; (**e**–**h**) with SD. OS_1_: SaH sludge; OS_2_: ArH sludge; OS_3_: Res sludge; OS_4_: Asp sludge).

To investigate the effect of reaction system temperature on the removal efficiency of petroleum hydrocarbons during the LSA/SD thermal washing process, four types of simulated oily sludge were treated under different temperature conditions (20–70 °C), while all other parameters were kept constant. Periodic sampling was conducted to analyze the petroleum hydrocarbon content. Figure 6a–h illustrates the time-dependent trends in petroleum hydrocarbon residue for the four types of simulated oily sludge under various temperature conditions, both with and without SD.

As observed from Figure 6a–d, in the process where LSA solution is used alone for the thermal washing of oily sludge, the removal amounts of SaH and ArH at 30 °C and 70 °C are similar and reach their maximum. In contrast, the removal of Res peaks at 30 °C, while Asp exhibit the highest removal at 70 °C. The maximum removal amounts of SaH, ArH, Res, and Asp are 216.476 mg, 128.762 mg, 96.916 mg, and 17.613 mg, respectively. During the removal of simulated oily sludge for SaH and ArH, the removal amount first increases, then decreases, followed by another increase. This phenomenon could be attributed to the initial temperature rise reducing the oily sludge’s viscosity [24], facilitating the washing of lower-density light oil hydrocarbons (SaH and ArH). Subsequently, as the temperature continues to rise, the hydrogen bonding between LSA molecules and water weakens, increasing the hydrophilicity and reducing the exposure of hydrophobic groups [25], thereby decreasing the interaction with petroleum hydrocarbons and resulting in a drop in removal efficiency. However, further temperature elevation enhances thermal motion between molecules, making the light oil more easily removed, while increasing the chances of LSA contacting the light oil, leading to a higher removal amount [26]. A similar trend was observed for Res, but lower temperatures favored its removal. At higher temperatures, the Res may fuse with other heavier components, reducing removal efficiency, while Asp significantly benefit from elevated temperatures, leading to a substantial increase in removal. In the process of thermal washing with the synergistic action of SD and LSA solutions, the removal amounts of SaH, ArH, Res, and Asp all increase with temperature. SaH and ArH reach equilibrium at 50 °C, while Res and Asp stabilize at 60 °C. The final removal amounts of petroleum hydrocarbons were 240.168 mg, 157.444 mg, 120.281 mg, and 30.370 mg, respectively. This indicates that the addition of SD alters the temperature response characteristics of each component. The oxidative effect of SD promotes the degradation of ArH, Res, and Asp, thus improving overall removal efficiency. Regarding reaction time, as shown in Figure 6a–d, when LSA is used alone, the removal of SaH, ArH, Res, and Asp reaches equilibrium within 60 min. However, with the addition of SD, as shown in Figure 6e–h, the removal of SaH still reaches equilibrium within 60 min, while the removal of ArH, Res, and Asp requires up to 80 min due to the oxidative degradation effect of sodium persulfate. Without SD, SaH and ArH emulsify easily with LSA and react quickly, while the emulsification of Res and Asp is more difficult, leading to an earlier termination of the reaction [21]. With the addition of SD, its oxidative action promotes the removal of petroleum components. Unlike the rapid equilibrium achieved by the easily emulsified SaH, the equilibrium points for the removal of ArH, Res, and Asp take longer, likely due to the time required for the oxidative degradation of large-molecule hydrocarbons into smaller ones.

According to the Arrhenius equation, the reaction rate constant *k* is given by:

(4) $$k={Ae}^{-\left(\frac{E}{RT}\right)}$$

Taking the logarithm of both sides, Equation (5) is obtained:

(5) $$\log\left(k\right)=\log\left(A\right)-\frac{E}{RT}$$

By substituting Equation (18) into Equation (4) and taking the logarithm of both sides, Equation (6) can be derived:

(6) $$\log\left(k\right)=\log\left(\frac{dw}{dt}\right)-a_{n}\times \left[{OH}^{-}\right]-b_{n}\left[LSA\right]-c_{n}\times \left[SD\right]$$

When SD is not added, Equation (19) is transformed into Equation (7):

(7) $$\frac{d_{w_{\left(PHy\right)}}}{d_{t}}=A\times e^{-\left(\frac{E}{RT}\right)}\times {\left[{OH}^{-}\right]}^{a}\times {\left[LSA\right]}^{b}$$

Substituting Equation (7) into Equation (5) and taking the logarithm of both sides yields Equation (8):

(8) $$\log\left(k\right)=\log\left(\frac{dw}{dt}\right)-a_{n}\times \left[{OH}^{-}\right]-b_{n}\left[LSA\right]$$

**Figure 7:**
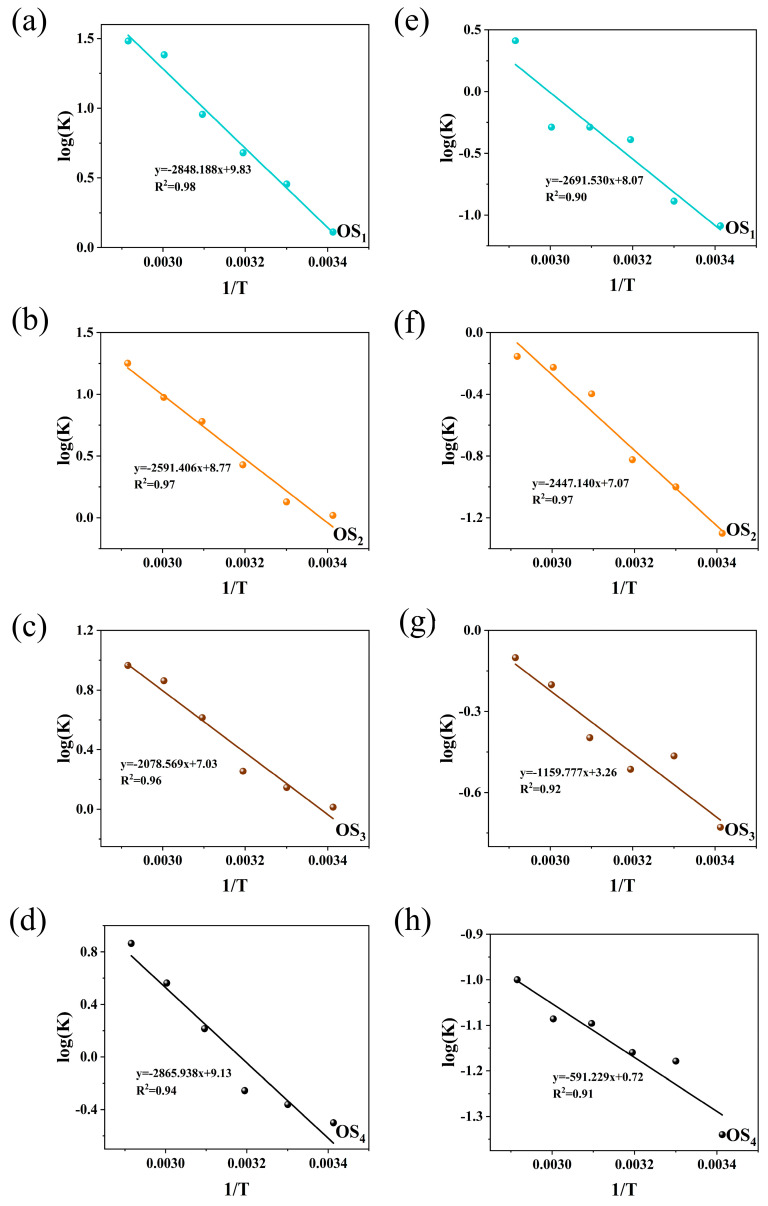
Linear relationship between log (d*w*/d*t*) and 1/T by hot washing with LSA/SD ((**a**–**d**) without SD; (**e**–**h**) with SD. OS_1_: SaH sludge; OS_2_: ArH sludge; OS_3_: Res sludge; OS_4_: Asp sludge).

The reaction order values for *a*_n_, *b*_n_, and *c*_n_, along with the optimal concentrations of hydroxide ions, LSA, and SD obtained from experiments, are substituted into Equations (6) and (8) to calculate the corresponding log(*k*) values for the four types of petroleum hydrocarbons. Through linear regression analysis, the linear relationship between log (*k*) and 1/T for the four types of simulated oily sludge, with and without sodium persulfate in the reaction, can be determined, as shown in Figure 7a–h.

As shown in Figure 7, the effect of temperature on the removal of heavy petroleum components, particularly Asp, becomes significantly more pronounced. The removal amount of petroleum hydrocarbons increases substantially with rising temperature. The removal rate follows the order of Asp, Res, ArH, and SaH. Using univariate linear regression analysis and based on Equation (6), the activation energy (*E*) and pre-exponential factor (*A*) for the reaction process were calculated. Under the thermal washing conditions without the addition of SD, the linear relationship between log(*K*) and *1/T* for SaH, ArH, Res, and Asp is shown in Figure 7a–d, with activation energies of 23,679.835 J/mol, 21,544.950 J/mol, 17,251.223 J/mol, and 23,827.409 J/mol, respectively. After the addition of SD, the linear relationship between log (*K*) and 1/T is shown in Figure 7e–h, where the activation energies of these petroleum components significantly decrease to 22,377.380 J/mol, 20,345.522 J/mol, 9642.386 J/mol, and 4915.478 J/mol, respectively. This indicates that sodium persulfate has a degradative effect on SaH, ArH, Res, and Asp, thereby enhancing the solubilization and curling effects of lignosulfonate-based alkyl agents on petroleum hydrocarbons. Asp exhibit the greatest decrease in activation energy, which may be attributed to their being the largest and most complex molecules in asphalt. Asp are composed of various high-molecular-weight hydrocarbons and their derivatives and may contain more unsaturated bonds (such as double or triple bonds), active functional groups (such as hydroxyl or carboxyl groups), and trace metal elements such as N, O, S, vanadium (V), nickel (Ni), iron (Fe), sodium (Na), calcium (Ca), and copper (Cu). These unsaturated bonds and functional groups are highly reactive in chemical reactions, making them prone to oxidation [13]. In contrast, ArH and Res have lower molecular weights and less complexity than Asp, containing only unsaturated benzene ring structures, which confer greater overall stability and make them less susceptible to oxidation. The degradation of SaH requires higher activation energy, possibly because the carbon atoms in SaH are connected by single bonds, which are relatively stable and less likely to undergo bond breakage or rearrangement. Further analysis of the changes in pre-exponential factors shows that, in the absence of SD, the pre-exponential factors for SaH, ArH, Res, and Asp are 9823.282, 2565.444, 1344.860, and 175.774, respectively. After the addition of SD, these values decreased to 8960.870, 1257.231, 29.089, and 0.785, respectively. These data further corroborate the positive impact of SD on the oxidation reaction kinetics of petroleum hydrocarbons.

Therefore, under the thermal washing process using LSA alone, the oxidation kinetic models for SaH, ArH, Res, and Asp can be represented by Equations (9) to (12), respectively.

(9) $$\frac{dw}{dt}=9.823\times 10^{3}\times e^{-\left(\frac{23679.835}{RT}\right)}\times {\left[{OH}^{-}\right]}^{0.110}\times {\left[SL\right]}^{1.59}$$

(10) $$\frac{dw}{dt}=2.565\times 10^{3}\times e^{-\left(\frac{21544.950}{RT}\right)}\times {\left[{OH}^{-}\right]}^{0.104}\times {\left[SL\right]}^{1.780}$$

(11) $$\frac{dw}{dt}=1.344\times 10^{3}\times e^{-\left(\frac{17251.223}{RT}\right)}\times {\left[{OH}^{-}\right]}^{0.188}\times {\left[SL\right]}^{1.780}$$

(12) $$\frac{dw}{dt}=0.176\times e^{-\left(\frac{23827.409}{RT}\right)}\times {\left[{OH}^{-}\right]}^{0.068}\times {\left[SL\right]}^{2.77}$$

After the addition of SD, the oxidation kinetic equations for SaH, ArH, Res, and Asp are expressed as Equations (13) to (16):

(13) $$\frac{dw}{dt}=8.961\times 10^{3}\times e^{-\left(\frac{22377.380}{RT}\right)}\times {\left[{OH}^{-}\right]}^{0.058}\times {\left[SL\right]}^{0.976}\times {\left[SD\right]}^{0.203}$$

(14) $$\frac{dw}{dt}=1.257\times 10^{3}\times e^{-\left(\frac{20345.522}{RT}\right)}\times {\left[{OH}^{-}\right]}^{0.064}\times {\left[SL\right]}^{1.252}\times {\left[SD\right]}^{0.253}$$

(15) $$\frac{dw}{dt}=29.089\times e^{-\left(\frac{9642.386}{RT}\right)}\times {\left[{OH}^{-}\right]}^{0.092}\times {\left[SL\right]}^{1.289}\times {\left[SD\right]}^{0.451}$$

(16) $$\frac{dw}{dt}=0.785\times e^{-\left(\frac{4915.478}{RT}\right)}\times {\left[{OH}^{-}\right]}^{0.1}\times {\left[SL\right]}^{2.789}\times {\left[SD\right]}^{0.703}$$

From the analysis of the kinetic models of the four types of oily sludge, with and without SD, it can be observed that after the addition of SD, the reaction orders of [*OH*^−^] for SaH, ArH, and Res show a decreasing trend to varying degrees, with the reaction order approaching zero. This suggests that, in the LSA/SD thermal washing system, the reaction rate is nearly independent of [*OH*^−^]. This result is likely due to the fact that SD oxidation of SaH, ArH, and Res requires more time, causing a reduction in the reaction rate.

In contrast, for Asp, the reaction order of [*OH*^−^] shows an increasing trend, indicating that the reaction rate in the LSA/SD thermal washing system is dependent on [*OH*^−^]. This suggests that the oxidation degradation of Asp in the LSA/SD system requires a more alkaline environment, where basic conditions accelerate the oxidation degradation and emulsification of Asp. In both the kinetic models, with and without SD, the reaction order of LSA remains relatively unchanged, indicating that LSA does not participate in the chemical reactions and its effect remains consistent across the four petroleum hydrocarbon types. Therefore, it can be inferred that LSA primarily plays a role in emulsification and solubilization.

### 2.2. Infrared Spectroscopic Analysis of Oil Sludge Thermally Washed with SD/LSA

#### 2.2.1. Reaction Between LSA and SD

**Figure 8:**
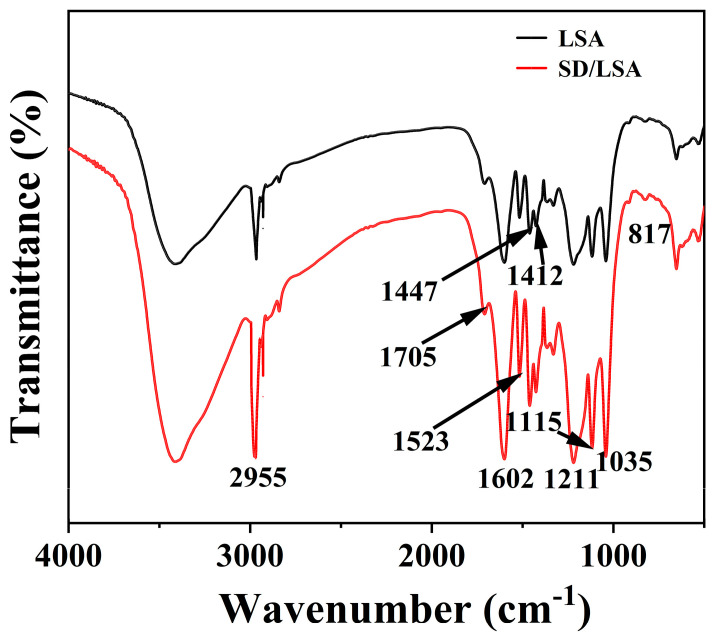
Infrared spectra of LSA before and after reaction with SD.

The infrared spectra of LSA before and after the reaction with SD are shown in Figure 8. The stretching vibration peaks of unconjugated carbonyl and conjugated carbonate from the carboxyl group (1705 cm^−1^) and ketone group (1602 cm^−1^) in lignin, as well as the C=O stretching vibration peak of aryl ketones (1523 cm^−1^), are evident. The peaks at 1523 cm^−1^, 1447 cm^−1^, and 1412 cm^−1^ correspond to the vibrational modes of the aromatic skeleton structure in lignin [27]. The peaks at 1115 cm^−1^ and 817 cm^−1^ represent the syringyl (S) and guaiacyl (G) units of lignin, respectively, indicating that the lignin structure of LSA primarily consists of S and G units [28]. The bands at 1211 cm^−1^ and 1035 cm^−1^ are attributed to the asymmetric and S=O stretching vibrations of SO_3_^2−^, respectively. These characteristic signals are clearly observed in the LSA both before and after the reaction, indicating that the sulfonate groups remain unaffected throughout the process [29]. The strong peak at 2955 cm^−1^ suggests that the methylene and methyl structures were not damaged. No new functional groups were detected, nor was the disappearance of any functional group observed, strongly indicating that no significant chemical reaction occurred between LSA and SD under optimal thermal washing conditions. This conclusion aligns with the aforementioned oxidation kinetics results.

#### 2.2.2. Reaction of Petroleum Hydrocarbons with LSA and SD

**Figure 9:**
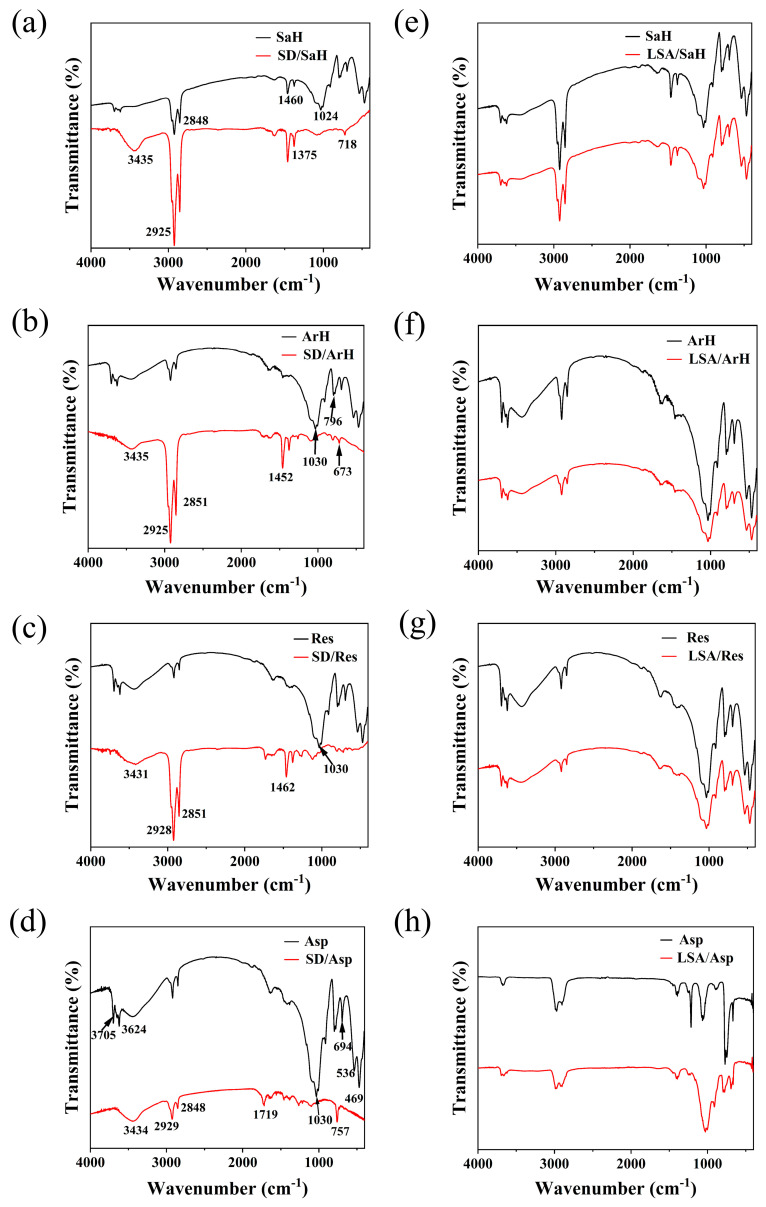
Infrared spectra before and after reaction of petroleum hydrocarbons under hot washing conditions ((**a**–**d**) SD with petroleum hydrocarbons; (**e**–**h**) LSA with petroleum hydrocarbons).

The infrared spectra of SD and LSA before and after reacting with the four types of petroleum hydrocarbons are shown in Figure 9. The C-H stretching vibrations of -CH_2_- and CH_3_ in SaH appear in the range of 3000–2800 cm^−1^. After treatment with LSA, as shown in Figure 9e, the peak shape of SaH remains unchanged, indicating that no chemical reaction occurred between SaH and LSA molecules [30]. However, after the reaction with SD, two prominent sharp absorption peaks appear at 2925 cm^−1^ and 2848 cm^−1^, as shown in Figure 9a, suggesting that SD effectively oxidized and degraded the SaH, resulting in the generation of more -CH_2_- and -CH_3_ groups. The bending vibration peaks of -CH_3_ and -CH_2_- at 1460 cm^−1^ and 1375 cm^−1^ are present in both SaH and SD/SaH, with an enhanced signal in SD/SaH, indicating that upon activation by sodium persulfate (SD), sulfate radicals (SO_4_^−^•) and hydroxyl radicals (•OH) catalyze the oxidative degradation of long-chain saturated hydrocarbons (SaH) into their short-chain analogs or facilitate the conversion of cyclic saturated hydrocarbons (SaH) into linear saturated hydrocarbons (SaH) through electron transfer mechanisms. Concurrently, the observed enhancement in -CH_2_- groups can be attributed to the generation of a greater number of olefinic hydrocarbons, exemplified by those possessing double bonds such as CH_2_=CH_2_, via hydrogen abstraction processes mediated by SO_4_^−^• and •OH [30]. Meanwhile, in Figure 9a–d, the infrared spectra of the four oil components after reaction show varying degrees of enhancement in the -OH peak at 3431 cm^−1^, indicating that during the oxidative cleavage process initiated by sodium persulfate (SD), the saturated hydrocarbons (SaH), aromatic hydrocarbons (ArH), resins (Res), and asphaltenes (Asp) all underwent oxidative degradation. This resulted in a decrease in the hydrophobicity and an increase in the hydrophilicity of the oil components. Notably, a broad strong absorption peak at 1024 cm^−1^, corresponding to the stretching vibration of the C-O bond, is present in SaH but significantly weakened in SD/SaH, reflecting that ether compounds were oxidized and degraded during the thermal washing process, possibly converting into alcohols [30]. This is further evidenced by the significant enhancement of the hydroxyl stretching vibration peak at 3435 cm^−1^. For ArH, as shown in Figure 9b,f, characteristic peaks for -OH antisymmetric stretching vibrations (alcohols or phenols) at 3435 cm^−1^, -CH_2_- and -CH_3_ asymmetric and symmetric stretching vibrations at 2925 cm^−1^ and 2851 cm^−1^, C=C stretching at 1452 cm^−1^, C-O stretching at 1030 cm^−1^, and benzene ring substitution peaks in the 670–830 cm^−1^ region are observed [31]. After the reaction between LSA and ArH, as seen in Figure 9f, the peak shape remains similar, indicating no significant chemical reaction occurred. However, in the infrared spectrum of SD/ArH (Figure 9b), the peak intensity at 3435 cm^−1^ is significantly enhanced, suggesting the breaking of ether bonds in ArH and the formation of alcohols or phenols. Peaks at 2925 cm^−1^ and 2851 cm^−1^ also show significant enhancement, indicating that after oxidation and degradation, ArH contained more methyl and methylene groups, confirming that they were degraded into smaller molecules. The sharper C=C stretching peak at 1452 cm^−1^ in SD/ArH indicates the destruction of long-chain ArH ether structures, resulting in smaller ArH molecules. The weakened C-O stretching peak at 1030 cm^−1^ further confirms the effective degradation of ether compounds [32]. The benzene ring substitution peaks in the 670–830 cm^−1^ range also weaken in SD/ArH, supporting the conclusion of ether degradation. The infrared spectrum of Res is similar to that of SaH and ArH, showing characteristic peaks for -OH, C-H, C=C, and C-O bonds. After LSA treatment, as seen in Figure 9g, the peak shapes remain similar, indicating that no significant chemical reaction occurred between LSA and Res. However, after SD treatment, as shown in Figure 9c, the peaks for -OH, C-H, and C=C in SD/Res are enhanced, while the C-O peak is weakened, suggesting that ether compounds in Res were also oxidized and degraded. The resin components include both SaH and ArH. The infrared spectra of Asp exhibit similar patterns to those of Res, as seen in Figure 9d,h, confirming the complexity and similarity in the chemical composition of these two substances. In summary, LSA did not undergo chemical reactions with any of the four petroleum hydrocarbon types, while SD effectively oxidized and degraded the benzene rings and long-chain ether compounds in SaH, ArH, Res, and Asp.

#### 2.2.3. Infrared Spectral Analysis of SD Before and After Reaction with Clean Soil

**Figure 10:**
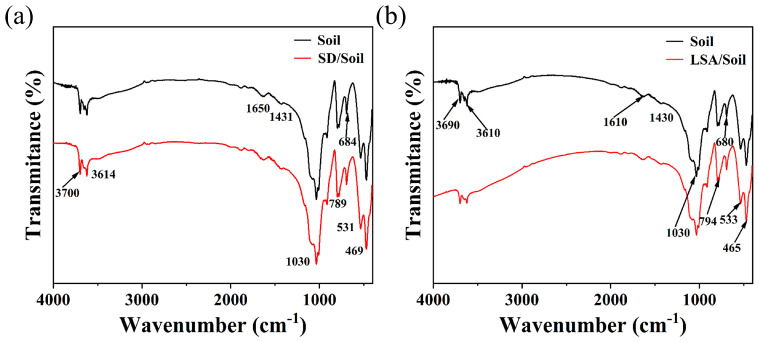
Infrared spectra before and after reaction of petroleum hydrocarbons under hot washing conditions ((**a**) SD with soil; (**b**) LSA with soil).

Figure 10 shows the infrared spectra of clean soil before and after thermal washing with LSA and SD. The absorption peak at 3690 cm^−1^ corresponds to the external -OH vibration of kaolinite’s octahedral coordination, while the peak at 3610 cm^−1^ represents the -OH stretching vibration at the interface between the tetrahedral and octahedral layers of the kaolinite structure unit, indicating the presence of kaolinite in the clean soil. Peaks at 1610 cm^−1^ and 1430 cm^−1^ correspond to the stretching vibrations of NO_3_ and NO_2_, suggesting the presence of nitro compounds in the clean soil. The band between 1650 and 1600 cm^−1^ corresponds to nitroso groups, while peaks at 1093 cm^−1^, 794 cm^−1^, 533 cm^−1^, and 465 cm^−1^ are associated with Si-O stretching and bending vibrations, confirming that the clean soil primarily consists of SiO_2_ [33]. The Al-OH stretching vibration peak at 1030 cm^−1^ indicates the presence of aluminum elements in the clean soil. The carbonate (CO_3_^2−^) bending vibration peak at 680 cm^−1^ suggests that the soil contains carbonates. The infrared spectra of soil after thermal washing with LSA and SD are consistent with those of clean soil, indicating that no chemical reactions occurred between LSA, SD, and the soil.

### 2.3. Physicochemical Characterization of Oil Hydrocarbon Components with SD/LSA

**Figure 11:**
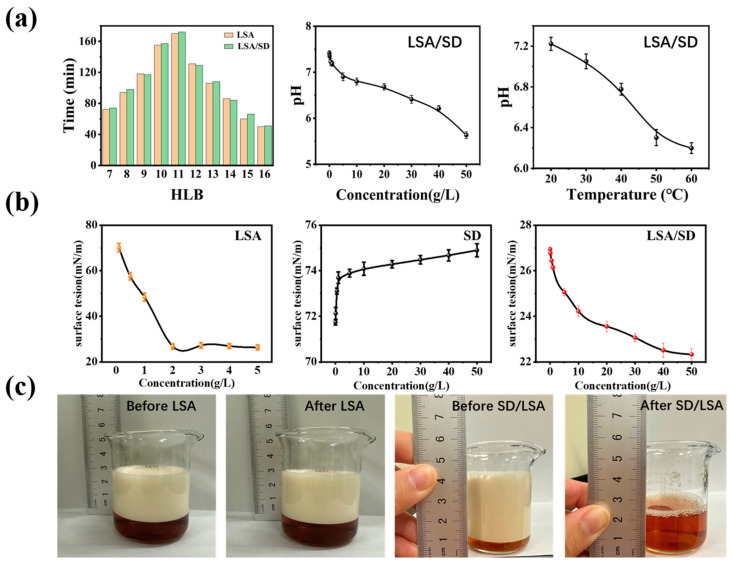
Characteristics analysis of sodium lignosulfonate alkylate and sodium persulfate reaction ((**a**) HLB and pH; (**b**) surface tension; (**c**) foam stability).

The hydrophilic–lipophilic balance (HLB) and pH values of the LSA solution and its mixture with SD were measured, as shown in Figure 11a. The HLB values before and after the reaction did not exhibit significant changes, indicating that under the given conditions, SD did not cause substantial oxidative degradation of LSA, thereby maintaining its original hydrophilic–lipophilic balance properties. From the observed pH changes, it was found that as SD concentration and reaction temperature increased, the pH of the solution showed a downward trend, though not a pronounced one. This could be attributed to the fact that when SD is thermally activated, it can generate sulfate radicals (SO₄^−^•), which promote the formation of H⁺ or other species that lower the solution’s pH [13]. The surface tension measurements of SD, LSA, and their mixture are shown in Figure 11b. The surface tension of the SD solution increased slightly with concentration, but this increase was not significant, suggesting that SD itself has a minimal impact on the solution’s surface tension. The increase from 71.828 mN/m to 74.636 mN/m is in accordance with the theory in colloid and interface chemistry, which states that inorganic salts are incapable of reducing the surface tension of solutions. In contrast, the surface tension of the LSA solution decreased with increasing concentration, indicating that LSA has the effect of reducing the surface tension of solutions, which is consistent with the properties of surfactants in colloid and interface chemistry. This demonstrates that LSA is an effective surfactant for hot washing. When the concentration of LSA reached 2.0 g/L, its surface tension reached a minimum, with subsequent changes remaining stable at a surface tension value of 29.874 mN/m., For the LSA-SD mixed solution, its surface tension is not only influenced by the surface tension-reducing effect of sodium lignosulfonate alkyl (LSA) but also exhibits an even lower surface tension, reaching a minimum value of 22.336 mN/m. This observation suggests that although sodium persulfate (SD) itself does not possess the capability to reduce surface tension, it can enhance the surface tension-lowering efficacy of the sodium lignosulfonate alkyl compound. This enhancement may be attributed to the formation of a diffuse double layer structure by ionic surfactant molecules in aqueous solutions. Upon the addition of sodium persulfate (SD), the increased concentration of counterions compresses the double layer of the surfactant, leading to a reduction in the electrostatic repulsion between the ionic hydrophilic head groups of the surfactant. This structural change allows the surfactant molecules to pack more densely on the solution surface, facilitating micelle formation and making it easier for the surfactant molecules to aggregate, thereby reducing the surface tension of the system [9]. Regarding foam stability, as shown in Figure 11c, the addition of SD resulted in denser foam with a lighter color compared to the LSA solution alone, and SD exhibited some defoaming ability. From Figure 11c and Equation (17), it can be concluded that SD had an inhibitory effect on the foam stability of the LSA solution. Although SD enhanced the foaming capacity of LSA (increasing the foaming capacity from 170.0% to 246.0%), it significantly reduced foam stability. This could be because the addition of SD altered the ionic environment and the intermolecular interactions between surfactant molecules in the solution, thereby affecting foam stability and persistence.

(17) $$Fa\left(\%\right)=\frac{H_{1}-H_{0}}{H_{0}}*100\%$$

where *Fa* is the foaming capacity, *H*_0_ is the initial solution height, and *H*_1_ is the total height of the solution and initial foam.

## 3. Materials and Methods

### 3.1. Materials and Reagents

Oily sludge was obtained from the Tahe Oilfield (Tahe, Xinjiang, China) and clean soil was obtained from the Guangxi University (Nanning, Guangxi, China). Toluene, n-hexane, dichloromethane, chloroform, anhydrous ethanol, sodium persulfate, lignosulfonate Alkylates, and sodium hydroxide were purchased from Aladdin Reagent Co., Ltd. (Shanghai, China). All chemicals used were of analytical grade. The separation of crude oil fractions (saturated hydrocarbons, aromatic hydrocarbons, resins, and asphaltenes) was carried out according to the Chinese Industry Standard SY/T 5119-2016 [34].

### 3.2. Preparation of Simulated Oily Sludge

Petroleum mainly comprises four components: saturated hydrocarbons (SaH), aromatic hydrocarbons (ArH), resins (Res), and asphaltenes (Asp). These are insoluble in water but soluble in non-polar solvents. SaH, linked by single carbon–carbon bonds, are low-density, highly non-polar, and stable, yet can undergo substitution. ArH, featuring benzene rings, have densities below water, with para-isomers showing the highest melting points, and undergo electrophilic substitution. Resins are poorly fluid, with a relative density > 1, short C–C bonds, and are polar but chemically unstable. Asphaltenes, complex mixtures of high-molecular-weight hydrocarbons and non-metal derivatives, also have a relative density > 1, are difficult to melt, and are chemically stable. Based on these properties, four simulated oil sludges were prepared for each component.

**Figure 12:**
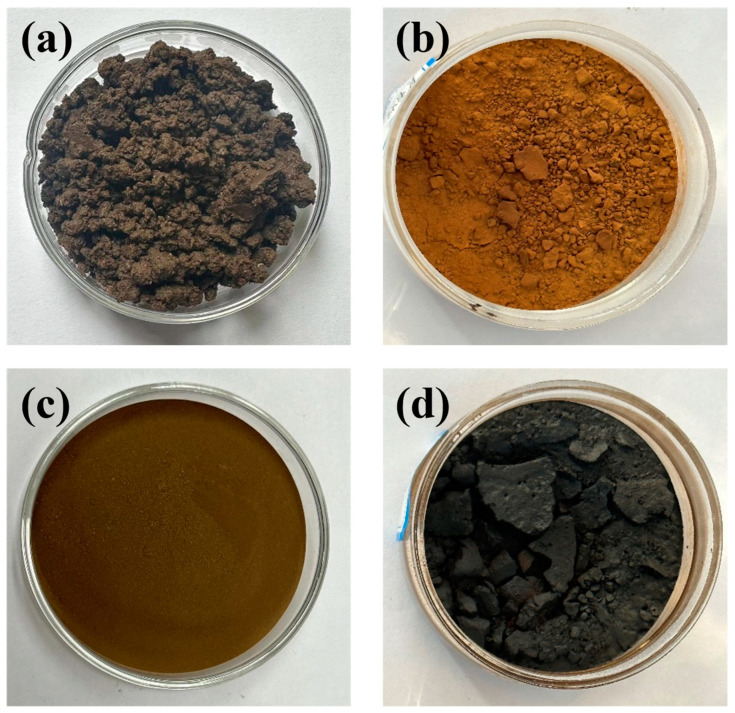
The physical image of simulated oily sludge ((**a**) saturated hydrocarbon; (**b**) aromatic hydrocarbon; (**c**) colloid oil hydrocarbon; (**d**) asphaltene hydrocarbon).

The preparation of simulated oily sludge was based on the method of Zhao et al. [35]. with slight modifications, and the specific steps were as follows: Oily sludge from Taha Oilfield in Xinjiang was processed using a Soxhlet extractor for azeotropic distillation with toluene and water at 130 °C, separating the sludge into three phases: oil, water, and sediment. The oil fraction was vacuum-evaporated and left to stand in n-hexane, allowing the precipitation of Asp. The remaining oil fractions were finely separated into SaH, ArH, and Res through a silica gel and neutral alumina chromatographic column using n-hexane, dichloromethane, anhydrous ethanol, and chloroform as eluents. The separated four oil fractions were then mixed with clean campus soil (dissolved in appropriate solvents to ensure even distribution). After natural solvent evaporation, four types of simulated oily sludge were obtained for subsequent experiments. Figure 12 shows the physical appearances of the simulated oily sludge.

### 3.3. Oxidative Degradation Experiment of Simulated Oily Sludge

The oxidative degradation experiments of simulated oily sludge were conducted with slight modifications to the methods reported by Nie and Shing et al. [36,37]. The effects of key parameters, including the pH of the washing solution (5, 7, 9, 10, 11, 12, 13, 14), LSA concentrations (0.5, 1.0, 1.5, 2.0, and 2.5 g/L), SD concentrations (0.5%, 1.0%, 2.0%, 4.0%, and 5.0%), and reaction temperatures (20 °C, 30 °C, 40 °C, 50 °C, 60 °C, and 70 °C), on the oil removal efficiency of four types of simulated oily sludge were systematically investigated over varying washing durations (10 min, 20 min, 30 min, 50 min, 60 min, 80 min, and 120 min). Constant experimental conditions included a liquid-to-solid ratio of 25:1 and a stirring speed of 150 rpm. Following the washing process, the solutions were filtered, and the oily sludge was dried at 40 °C. The pH of the reaction system was adjusted using 2.0 mol/L sodium hydroxide solution and hydrochloric acid solution to ensure precise control of the initial pH value. The dried oily sludge was ground into powder, and hydrocarbons were extracted using chloroform. The concentrations of different hydrocarbons were quantified using the UV spectrophotometer (Specord 250 Plus, Analytik Jena, Berlin, Germany). The experiments were performed in triplicate, and the average values were recorded. The removal of petroleum hydrocarbons (W_PHy_) was calculated according to Equation (18).

(18) $$W_{PHy}=w\times m_{OS}-C\times V$$

where *W_PHy_* represents the amount of petroleum hydrocarbons removed (mg), *w* denotes the oil content percentage (%) per gram of oily sludge, *m_OS_* refers to the mass of the oily sludge (mg), *C* is the concentration of petroleum hydrocarbons (%), and *V* is the volume of the extraction solution (mL).

The traditional oxidative degradation Equation (19) was employed to explore the mechanism of oxidative degradation of petroleum hydrocarbons by LSA and SD [37].

(19) $$\frac{d_{W_{\left(PHy\right)}}}{d_{t}}=A\times e^{-\left(\frac{E}{RT}\right)}\times {\left[{OH}^{-}\right]}^{a}\times {\left[LSA\right]}^{b}\times {\left[SD\right]}^{c}$$

In this equation, *W_PHy_* represents the amount of petroleum hydrocarbons removed (mg), *t* refers to the washing time (min), d*w*_(*PHy*)_/d*t* denotes the degradation reaction rate, *A* is the pre-exponential factor, *E* is the activation energy (kJ/mol), *R* is the universal gas constant (8.314 J·mol^−1^·K^−1^), and *T* is the reaction temperature (*K*). [*OH*^−^] indicates the hydroxide ion concentration (mol/L), [*LSA*] is the concentration of lignosulfonate alkyl compounds (g/L), and [*SD*] is the concentration of sodium persulfate (%). The exponents *a*, *b*, and *c* represent the reaction orders of hydroxide ions, lignosulfonate alkyl compounds, and sodium persulfate, respectively.

### 3.4. Physicochemical Properties of the Reaction Between Sodium Persulfate and Sodium Lignosulfonate Alkylate

#### 3.4.1. Infrared Characterization

Fourier transform infrared spectroscopy (FTIR, VERTEX 70 Bruker, Berlin, Germany) was used to investigate the interactions between sodium persulfate and alkylated lignosulfonate, petroleum hydrocarbons (SaH, ArH, Res, Asp), and clean soil. The sample preparation process was as follows: lignosulfonate Alkylates was mixed with sodium persulfate under optimized conditions. The alkylated lignosulfonate was then extracted with methanol, rotary evaporated, and freeze-dried for further use. Sodium persulfate or lignosulfonate alkylates was reacted with petroleum hydrocarbons under optimized conditions. After solvent extraction and evaporation, the four types of petroleum hydrocarbons were prepared for testing. Sodium persulfate or lignosulfonate alkylates were also reacted with clean soil under optimized conditions. The reacted soil was subjected to repeated washing with distilled water and subsequently dried for further analysis. The changes in functional groups before and after the reaction were analyzed through infrared spectroscopy. The FTIR testing conditions were as follows [38]: potassium bromide was mixed with the prepared samples in a 100:1 ratio, with a spectral range of 4000–400 cm^−1^ and a resolution of 2 cm^−1^.

#### 3.4.2. Surface Tension Measurement

The surface tension of lignosulfonate surfactant, sodium persulfate, and their mixtures was measured using a surface/interfacial tension meter (K100, KRÜSS GmbH, Berlin, Germany). Sample preparation involved the accurate formulation of lignosulfonate surfactant solutions at concentrations of 0.1, 0.5, 1.0, 1.5, 2.0, 2.5, 3.0, 4.0, and 5.0 g/L, as well as sodium persulfate solutions at concentrations of 1.0%, 2.0%, 3.0%, 4.0%, and 5.0%. The critical micelle concentration of the lignosulfonate surfactant was determined, and at this concentration, each sodium persulfate solution was mixed sequentially with the lignosulfonate surfactant solution for further analysis.

#### 3.4.3. pH Measurement

The pH values of lignosulfonate surfactant/sodium persulfate mixtures at varying concentrations and temperatures were measured using a pH meter (pH, FiveEasy Plus, Shanghai, China). Sample preparation involved the formulation of a 2 g/L lignosulfonate surfactant solution. A specified mass of sodium persulfate was gradually added to the lignosulfonate surfactant solution to prepare mixtures with concentrations of 0.0, 0.1, 0.5, 1.0, 5.0, 10.0, 20.0, 30.0, 40.0, and 50.0 g/L for pH determination. Additionally, the pH of the 50.0 g/L sodium persulfate was measured at temperatures ranging from 20 to 60 °C.

#### 3.4.4. Foam Stability Assessment

Following the modified foaming method of Zhang et al. [39], 25 mL of 2.0 g/L LSA and LSA/SD solutions were separately measured, and foaming was carried out using a homogenizer for 1 min. The foaming and defoaming behavior were recorded after standing for 10, 30, and 60 min. The foaming properties of LSA solutions with and without SD at the same concentration were evaluated.

#### 3.4.5. Determination of HLB Value

The hydrophilic-lipophilic balance (HLB) values of lignosulfonate alkylated surfactants and their mixtures with sodium persulfate solutions were determined using the emulsification method as described by Chen [40]. The detailed procedures are outlined as follows: 5% sodium persulfate and 2.0 g/L lignosulfonate surfactant (SD-LSA) was determined using the emulsification method in a sealed cylinder with a volume of 100 mL. The experimental procedure began by preparing a series of standard oil samples with varying HLB values (7, 8, 9, 10, 11, 12, 13, 14, 15, and 16) using cottonseed oil (HLB = 6) and turpentine oil (HLB = 16). Subsequently, 20 mL of each standard oil sample with different HLB values and 20 mL of the SD-LSA solution were added sequentially to the cylinder. The cylinder was then vigorously shaken and allowed to stand until clear phase separation was observed. The time required for oil-water separation was recorded, and the HLB value of the SD-LSA mixed solution was determined based on the HLB value corresponding to the standard oil sample that exhibited the best emulsification performance.

## 4. Conclusions

In this study, sodium persulfate (SD) and lignosulfonate (LSA) were employed as reagents to explore the mechanism of thermal washing in oily sludge treatment. The results indicate that the combination of SD and LSA solution alters the interfacial tension between oily sludge particles and the aqueous phase, facilitating the dispersion and emulsification of oil contaminants. During the simulated oily sludge thermal washing process, SD did not oxidatively degrade LSA under certain conditions, and the radicals generated by SD were more effective at attacking organic molecules with the assistance of LSA, leading to faster degradation rates and higher removal efficiency of petroleum hydrocarbons. The activation energies of SaH, ArH, Res, and Asp were reduced from 23,679.835 J/mol, 21,544.950 J/mol, 17,251.223 J/mol, and 23,827.409 J/mol to 22,377.380 J/mol, 20,345.522 J/mol, 9642.386 J/mol, and 4915.478 J/mol, respectively. The synergistic effect of LSA and SD in enhancing the removal of petroleum hydrocarbons was significant. The findings provide valuable data and theoretical insights for optimizing oily sludge treatment processes.

## Data Availability

Data are contained within the article.
